# Enterohemorrhagic *Escherichia coli* outbreaks related to childcare facilities in Japan, 2010–2013

**DOI:** 10.1186/s12879-015-1259-3

**Published:** 2015-11-20

**Authors:** Atsuhiro Kanayama, Yuichiro Yahata, Yuzo Arima, Takuri Takahashi, Takehito Saitoh, Kazuhiko Kanou, Kunio Kawabata, Tomimasa Sunagawa, Tamano Matsui, Kazunori Oishi

**Affiliations:** Field Epidemiology Training Program, National Institute of Infectious Diseases, 1-23-1 Toyama, Shinjuku-ku, Tokyo 162-8640 Japan; Department of Global Infectious Diseases and Tropical Medicine, National Defense Medical College, Saitama, Japan; Infectious Disease Surveillance Center, National Institute of Infectious Diseases, Toyama, Shinjuku-ku, Tokyo 162-8640 Japan

**Keywords:** Enterohemorrhagic *Escherichia coli*, Non-O157, Childcare facility, Outbreak, Surveillance

## Abstract

**Background:**

Enterohemorrhagic *Escherichia coli* (EHEC) is an important cause of gastroenteritis in Japan. Although non-O157 EHEC infections have been increasingly reported worldwide, their impact on children has not been well described.

**Methods:**

We collected national surveillance data of EHEC infections reported between 2010 and 2013 in Japan and characterized outbreaks that occurred in childcare facilities. Per Japanese outbreak investigation protocol, faecal samples from contacts of EHEC cases were collected regardless of symptomatic status. Cases and outbreaks were described by demographics, dates of diagnosis and onset, clinical manifestations, laboratory data, and relation to specific outbreaks in childcare facilities.

**Results:**

During 2010–2013, a total of 68 EHEC outbreaks comprised of 1035 cases were related to childcare facilities. Among the 66 outbreaks caused by a single serogroup, 29 were serogroup O26 and 22 were O157; 35 outbreaks were caused by *stx1*-producing strains. Since 2010, the number of reported outbreaks steadily increased, with a rise in cases and outbreaks caused by *stx1*-producing O26. Of 7069 EHEC cases reported nationally in 2010–2011, the majority were caused by O157 (*n* = 4938), relative to O26 (*n* = 1353) and O111 (*n* = 195). However, relative to 69 cases of O157 (2 %) associated with childcare facility EHEC outbreaks, there were 131 (10 %) such cases of O26, and this trend intensified in 2012–2013 (O157, 3 %; O26, 24 %; O111, 48 %). Among family members of childcare facility cases, the proportion of cases that were symptomatic declined with age; 10/16 cases (63 %) aged 6 years or younger, 16/53 cases (30 %) 6–19 years old, 23/120 cases (19 %) 20–49 years old and 2/28 cases (7 %) 50 years or older were symptomatic. Thirty one of the 68 outbreaks (46 %) were classified as foodborne-related.

**Conclusions:**

Childcare facility EHEC outbreaks due to non-O157 serogroups, particularly O26 and O111, increased during 2010–2013. These facilities should pay extra attention to health conditions in children. As older family members of childcare facility cases appear to be less symptomatic, they should be vigilant about hand-washing to prevent further transmission.

## Background

Enterohemorrhagic *Escherichia coli* (EHEC) is known to cause diarrhea and hemorrhagic colitis in humans [1]. Vulnerable populations of EHEC infection include small children and the elderly. Severe cases are reported more often in children than in adults (under 65 years of age), and may include life-threatening hemolytic uremic syndrome (HUS) [2]. EHEC cases in outbreaks have predominantly been attributed to EHEC O157:H7; however, recent studies have reported an increased in the number of cases affected by non-O157 serogroups, including O26, O103, O111, and O145 [3]. Non-O157 *E. coli* strains have been isolated from patients in the USA [4], Europe [5, 6], Australia [7], New Zealand [8], and Japan [9]. Notably, non-O157 *E. coli* tended to affect younger children more often than O157 in the USA [4].

EHEC outbreaks in childcare facilities have been reported from several countries [10–19]. Higher rates of secondary transmission have been detected in EHEC O157 outbreaks among people with a median age of less than 6 years compared with those with a median age of 6–59 years [12]. Although information regarding non-O157 outbreaks in childcare settings have been limited, non-O157 infections seem to occur more frequently than O157 infections in childcare facilities [13–19]. However, the reasons remain unknown and there is no consensus regarding how these outbreaks should be controlled.

EHEC is a notifiable disease in Japan and approximately 4000 EHEC cases are reported annually in the country, including outbreaks associated with childcare facilities [16–19]. After being notified of a case by a physician, Local Public Health Centers (LPHCs) conduct active case-finding among contacts of case patients as described previously [20]. LPHC staff interview contacts about their health status, collect their faecal specimens (regardless of symptomatic status), and monitor their health status. Such protocol is designed to allow for rapid and timely reporting of outbreaks, including those associated with childcare facilities, through the National Epidemiological Surveillance of Infectious Diseases (NESID) system from each LPHC. In August 2013, an increase in the reported number of EHEC outbreaks in childcare facility settings was noted, especially after 2012. Although more than 2.2 million children attending childcare facilities are at potential risk of EHEC infection [21], the overall picture on outbreaks in these settings has yet to be determined.

We describe a recent increase in the number of reported EHEC outbreaks related to childcare facilities in Japan based on analysis of national surveillance data. We discuss the outbreaks in terms of number of outbreaks and cases, proportion of symptomatic cases among cases detected through active investigations, characteristics of isolated serogroups and *stx* types, and mode of transmission.

### Ethics

This study was conducted by the Act on Prevention of Infections and Medical Care for Patients with Infections in Japan. Thus, this study did not require approval from an ethics committee.

## Methods

### Data collection

EHEC infection is a notifiable disease in Japan, requiring an immediate report after diagnosis per “Act on Prevention of Infections and Medical Care for Patients with Infections”. Local governments collect and submit case-based data via NESID to the Ministry of Health, Labour and Welfare (MHLW). For the present study, the following data were extracted from NESID: dates of notification, age, sex, occupation, clinical manifestations, serogroup and *stx* type from isolated EHEC strains, suspected route of infection (foodborne, direct contact, both foodborne and direct contact, or unspecified, as indicated by the reporting physicians and LPHC), and relationship to a specific outbreak in a childcare facility.

A symptomatic case was defined as EHEC infection confirmed by culture and serological testing for anti-Shiga toxin (*stx*) antibody or Polymerase Chain Reaction (PCR), after the development of at least one gastrointestinal illness, such as watery diarrhea, abdominal pain or cramps, bloody diarrhea, or vomiting. The presence of Shiga toxin in stool samples was confirmed by enzyme immunoassay or PCR. Standardized methods for laboratory tests are recommended to LPHCs under the guidance of the National Institute of Infectious Diseases, based on guidelines from World Health Organization for *Escherichia coli* and *Klebsiella* [22]. An asymptomatic case was defined as an EHEC infection confirmed by the laboratory tests described above for symptomatic cases, detected by active case finding during outbreak investigation or from routine screenings required in designated occupations (e.g. food handler, nursery school employee).

### Childcare facility-related EHEC outbreaks

Per Japanese outbreak investigation protocol, after being notified of a case by a physician, LPHCs conduct active case-finding among contacts of case patients. When symptomatic EHEC cases are detected at a childcare facility, asymptomatic children at the same facility are screened by the laboratory tests described above. For our study, an outbreak at a childcare facility was defined as a cluster of at least two cases aged less than 7 years, attending the same childcare facility, affected by the same EHEC serogroup and *stx* type, reported from the same LPHC, and occurring within 3 weeks of diagnosis (approximately double the 10 days-incubation period). Outbreak-related cases were defined as cluster cases described above and cases with the same serogroup and *stx* as the cluster cases, and sharing the same information as cluster cases for any of the following: belonged to the same family, living in the same household, or epidemiologically linked to the same childcare facility or cluster. The suspected route of infection was indicated by the reporting physicians and LPHCs and categorized as either foodborne, direct contact, or unspecified.

All descriptive statistical analyses were based solely on the extracted NESID surveillance data. Based on the extracted data, cases reported during the period when an increase in EHEC outbreaks in childcare facilities was observed (2012–2013) were compared to an immediately prior period of the same length (2010–2011).

## Results

### Reported number of EHEC cases and childcare facility outbreaks

**Fig. 1:**
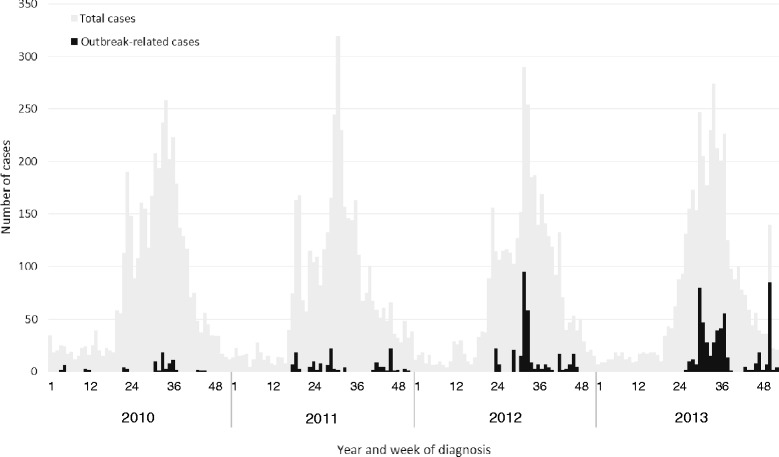
Epidemiological curve of EHEC cases in childcare facility-related outbreaks in Japan, 2010–2013. EHEC cases in childcare facility-related outbreaks are shown by week of diagnosis during 2010–2013 in black (indicated as Outbreak-related cases); total EHEC cases for the same period are shown in grey (indicated as Total cases)

Approximately 4000 EHEC cases were reported annually, peaking consistently at around epidemiological week 30 during 2010–2013 (Fig. 1). We identified 1035 outbreak-related cases among the 68 childcare facility outbreaks that occurred during this period. The number of outbreaks doubled during 2010–2013 and outbreak-related cases increased steadily over this period (Table 1). The increase in number of cases per outbreak in 2011–2013 was largely due to an increase in asymptomatic cases. However, the median number of symptomatic cases per outbreak remained the same (4, range: 1–15 for 2010–2011 and 1–47 for 2012–2013); moreover, in 2012–2013 there were 3 large scale outbreaks with more than 15 symptomatic cases compared to none in 2010–2011. Among the 68 outbreaks, 65 (96 %) outbreaks occurred between epidemiological weeks 22 and 50.

**Table 1:** Childcare facility-related EHEC outbreaks, 2010–2013, Japan. All outbreaks listed below in the table refer to childcare facility-related outbreaks

	Total	2010	2011	2012	2013
Total no. reported cases	15,887	4134	3940	3768	4045
No outbreaks	68	9	12	15	32
No. outbreak-related cases	1035	77	141	304	513
No. cases per outbreak	15.2	8.6	11.8	20.3	16.0
No. asymptomatic cases per outbreak	9.4	3.7	6.8	14.8	9.6
No. Hemolytic Uremic Syndrome cases in outbreaks	4	0	1	2	1

Majority of EHEC cases in 2010–2013 were caused by six serogroups (O26, O157, O111, O103, O121 and O145). While O157 was responsible for the majority of EHEC cases with a single serogroup and *stx1* and/or *stx2* type identified (4938/7069), O26 was the major serogroup among childcare-facility outbreaks (131/218), with 1 % of O157 cases vs. 10 % of O26 cases being outbreak-related (Table 2). This contrast between O157 and O26 became greater in 2012–2013 (2 % and 24 %, respectively). There were no childcare facility outbreaks associated with O111 in 2010–2011, but 173 O111 outbreak-related cases were reported in 2012–2013. Moreover, the proportion of O111 outbreak-related cases reached 48 % in 2012–2013.

**Table 2:** Childcare facility outbreak-related cases, total number of EHEC cases, and proportion of cases that were childcare facility outbreak-related cases, by serogroup, 2010–2013, Japan. All outbreaks listed below in the table refer to childcare facility-related outbreaks

Serogroup	2010–2011			2012–2013		
	Outbreak cases (n)	Total cases (n)	Outbreak/Total (%)	Outbreak cases (n)	Total cases (n)	Outbreak/Total (%)
O157	69	4938	1 %	100	4075	2 %
O26	131	1353	10 %	417	1707	24 %
O111	0	195	0 %	173	364	48 %
O103, O121 or O145 ^a^	18	583	3 %	64	684	9 %
Total ^b^	218	7069	3 %	754	6830	11 %

^a^ excludes cases related to two O26/O103 co-infection outbreaks

^b^ excludes cases with serogroups other than O157, O26, O111, O103, O121, or O145, co-infections of more than one serogroup and infections with unknown serogroup or stx type (1005 cases in 2010–2011 and 983 cases in 2012–2013)

### Characteristics of cases related to childcare facility outbreaks

Among a total of 1035 outbreak-related cases, 484 (47 %) were male and the median age was 4 years (range: 0–88 years) (Table 3). A total of 761 (74 %) cases were cluster cases, i.e. pediatric cases who were attending the childcare facilities that had an outbreak. Of 761 cases, 329 (43 %) were symptomatic. The majority of the remaining outbreak-related cases were family members, and the majority of family members were aged 20–49 years (120 cases). In 2010–2011, older age groups were less likely to be symptomatic than other family members. For example, 60 % of siblings of cluster cases less than 7 years old and with no evidence of going to the same childcare facility were symptomatic, while all 16 family members over 50 years old were asymptomatic. This trend continued in 2012–2013, when this youngest age group remained more likely to be symptomatic (64 %) than cases 7–19 and 20–49 years of age (23 % and 10 %, respectively). During both 2010–2011 and 2012–2013, among family member cases, age was inversely associated with being symptomatic, with only 7 % of those 50 years and older being symptomatic during 2010–2013 (Table 3). Four HUS cases were reported during 2010–2013; all four were less than 7 years old and affected by O157 that had both *stx1* and *stx2* and recorded in three outbreaks in 2013. Based on the available information among symptomatic cases, the longest interval between the onsets of cases in these outbreaks was 18 days.

**Table 3:** Demographic characteristics of childcare facility EHEC outbreak-related cases, 2010–2013, Japan. All outbreaks listed below in the table refer to childcare facility-related outbreaks

	Total				2010–2011				2012–2013			
	Symptomatic (n)	Asymptomatic (n)	Total (n)	Symptomatic (%)	Symptomatic (n)	Asymptomatic (n)	Total (n)	Symptomatic (%)	Symptomatic (n)	Asymptomatic (n)	Total (n)	Symptomatic (%)
Male	197	287	484	(41 %)	54	46	100	(54 %)	143	241	384	(37 %)
Female	196	355	551	(36 %)	50	68	118	(42 %)	146	287	433	(34 %)
Age in years												
<7, childcare facility	329	432	761	(43 %)	75	66	141	(53 %)	254	366	620	(41 %)
<7, family	10	6	16	(63 %)	3	2	5	(60 %)	7	4	11	(64 %)
7–19, family	16	37	53	(30 %)	6	4	10	(60 %)	10	33	43	(23 %)
20–49, family	23	97	120	(19 %)	15	22	37	(41 %)	8	75	83	(10 %)
≧50, family	2	26	28	(7 %)	0	8	8	(0 %)	2	18	20	(10 %)
Others ^a^	13	44	57	(23 %)	5	12	17	(29 %)	8	32	40	(20 %)

^a^ Others include those who were not or not known to be family members of the child attending the childcare facility

### Serogroups and stx types of the isolated EHEC strains in the childcare facility outbreaks

Among outbreaks caused by a single serogroup (*n* = 66; excluding two O26/O103 co-infection outbreaks), the *stx1*-producing strain was the most predominant (35/66, 53 %) (Table 4); 19/66 (29 %) outbreaks were associated with both the *stx1*- and *stx2*-producing strains and 12/66 (18 %) outbreaks were associated with the *stx2*-producing strain. Considering both Serogroups and *stx* types, O26 *stx1* accounted for the largest proportion (26/66, 39 %), followed by O157 *stx1* and *stx2* (14/66, 21 %) and O157 *stx2* (7/66, 11 %). During 2012–2013, there was a greater increase in both the number of cases and outbreaks associated with O26 relative to those associated with O157, particularly for *stx1*-producing O26 (Table 4).

**Table 4:** Number of childcare facility EHEC outbreak-related cases by serogroups and *stx* types, 2010–2013, Japan. All outbreaks listed below in the table refer to childcare facility-related outbreaks

Serogroup	*stx* type	Total		2010–2011		2012–2013		Ratio of 2012–2013 values to 2010–2011 values	
		Case	Outbreak	Case	Outbreak	Case	Outbreak	Case	Outbreak
O157	*stx*1	11	1	11	1	0	0	0	0
	*stx*2 or *stx*1/2	158	21	58	9	100	12	1.7	1.3
	Total	169	22	69	10	100	12	1.4	1.4
O26	*stx*1	478	26	86	7	392	19	4.6	2.7
	*stx*2 or *stx*1/2	70	3	45	2	25	1	0.6	0.5
	Total	548	29	131	9	417	20	3.2	2.2
O111	*stx*1	44	3	0	0	44	3	-	-
	*stx*2 or *stx*1/2	129	3	0	0	129	3	-	-
	Total	173	6	0	0	173	6	-	-
O103, O121 or O145 ^a^	*stx*1	48	5	11	1	37	4	3.4	4
	*stx*2 or *stx*1/2	34	4	7	1	27	3	3.9	3
	Total	82	9	18	2	64	7	3.6	3.5
Total	*stx1*	581	35	108	9	473	26	4.4	2.9
	*stx*2 or *stx*1/2	391	31	110	12	281	10	2.6	0.8
	Total	972	66	218	21	754	45	3.5	2.1

^a^ excludes cases related to two O26/O103 co-infection outbreaks

### Mode of transmission

Among the 68 outbreaks, suspected transmission routes were direct contact (24 outbreaks, 35 %), both foodborne and direct contact (24 outbreaks, 35 %), foodborne (7 outbreaks, 10 %), and unspecified (13 outbreaks, 19 %).

## Discussion

In this study, we identified 68 EHEC outbreaks associated with childcare facilities in Japan from 2010 to 2013. Notably, the proportion of cases associated with outbreaks varied by serogroup (Table 2). In particular, the number of such outbreaks increased in 2012–2013. O26 *stx1* cases comprised the majority of cases related to such outbreaks (39 % of outbreaks) during the period and contributed to much of the increase. O111 cases were also associated with childcare facility outbreaks and nearly half of all reported O111 cases were related to such outbreaks that occurred during 2012–2013 (Table 2). O157, however, appeared to be much less associated with such outbreaks. Fortunately, *stx1*-producing O26 is known to be less severe than O157 [2]. In fact, all four reported HUS cases during the study period were among O157 infections and much of the increase in outbreak-related cases was associated with an increase in asymptomatic cases. Family members, particularly older persons, of cases that were attending childcare facilities were less likely to be symptomatic. To our knowledge, this is the first study to show profiles of childcare facility-related EHEC outbreaks in Japan.

These results suggest that certain non-O157 strains, such as O111 and O26, may be more likely to occur in childcare facility settings and could indicate transmission concerns in these settings, particularly given the presence of asymptomatic cases. In fact, direct contact was the suspected route of transmission of EHEC infections in 48 of the 68 outbreaks (direct contact in 24 outbreaks and both foodborne and direct contact in 24 outbreaks), relative to foodborne transmission suspected in 31 outbreaks (foodborne in 7 outbreaks and both foodborne and direct contact in 24 outbreaks). Majority of adult family member cases were asymptomatic, particularly those over 50 years of age. Taken together, we speculate that a sizable proportion of adolescent and adults were unaware of their EHEC infection, and could transmit the infection to other family members, including their children, before control measures could be undertaken. Thus, adolescents and adults in close contact with young children should be vigilant about hand-washing even if they do not notice any signs or symptoms of gastroenteritis themselves. In particular, as O26, the most frequent serogroup detected among childcare facilities, tends to be more asymptomatic than O157, asymptomatic cases may contribute to further spread, resulting in larger outbreaks.

As EHEC shedding is known to be relatively long lasting, earlier detection of events and rapid outbreak investigations are important in minimizing the number of infected cases and reducing potential further spread. While only few studies have assessed such information, long duration of shedding has been documented for several EHEC serotypes. The duration of shedding ranged from 15 to 46 days in an EHEC O26 outbreak in the USA [15]. Shedding periods of up to 71 days were found in an EHEC O145 outbreak in a Norwegian childcare center [23]. Furthermore, a sensitive detection method used in a German outbreak showed that shedding lasted up to 62 days in those with diarrhea or hemorrhagic colitis [24]. Moreover, some outbreaks in Japan were difficult to control quickly, taking a mean of 46.5 days (range: 32–63) after the onset of index cases, partly due to secondary infections [25], emphasizing the need for early detection to prevent transmission. Family members are also affected by childcare facility-related cases, and while the direction of transmission between family members and cases in childcare facilities is unknown, proper hand washing and hygiene practice are important in households.

While the O157 serogroup is known for its severity, the United States Department of Agriculture pointed out that awareness of non-O157 serogroups as a food safety concern has increased in the USA and added six non-O157 serogroups (O26, O45, O103, O111, O121, and O145) to the list of pathogens that should be considered to be of public health importance [26]. Indeed, while O26 serogroup is known to be relatively less severe, concerns regarding O26 are starting to emerge. For example, a novel type of O26 clone harboring the *stx2* gene was found in Germany, and has been spreading across Europe, the USA, and South America [5, 27–30]. These studies suggested that *stx2*-producing O26 might be a more virulent type, and could pose a threat to children in childcare facilities in Japan and in other countries. The *stx2*-producing strain of O157 can also result in severe outcomes, including HUS [2]. The second major serogroup in our study was *stx2*-producing EHEC O157, and indeed, all four HUS cases detected in our study were affected by *stx1* and *stx2*-producing O157.

A key limitation in our study is understanding the reason why childcare facility-related non-O157 outbreaks have increased, which remains unclear from national surveillance-based analysis. As the increase was associated with increase in asymptomatic cases, possibility of enhanced outbreak investigations at child care facilities exist. However, there was no change in policy for diagnostic practices or surveillance criteria. Moreover, while the median number of symptomatic cases per outbreak remained the same, there was an increase in large scale outbreaks in 2012–2013. In addition, while specific alerts regarding childcare facility-related EHEC outbreaks were distributed from MHLW to LPHCs in late August 2013, this notification was based on increased EHEC cases from NESID data; thus, it cannot explain the increase in the number of outbreaks in 2012 or the increase during the period up to August in 2013, when the number of outbreaks had already reached almost twice as many as that in 2012.

## Conclusions

In Japan, the reported frequency of EHEC outbreaks and cases related to childcare facilities, especially those with non-O157 strains, have been increasing. Once infections with non-O157 serogroups in childcare facilities are recognized, family members should be vigilant about hand washing to prevent further transmission. Early detection and response is required to minimize the extent of outbreaks, and further investigation into the potential reasons of the increase in such outbreaks is warranted.

## Abbreviations

EHEC: Enterohemorrhagic *Escherichia coli*
HUS: Hemolytic uremic syndrome
*stx*: Shiga toxin
NESID: National Epidemiological Surveillance of Infectious Diseases
MHLW: Ministry of Health Labour and Welfare
